# The BioDICE Taverna plugin for clustering and visualization of biological data: a workflow for molecular compounds exploration

**DOI:** 10.1186/1758-2946-6-24

**Published:** 2014-05-13

**Authors:** Antonino Fiannaca, Massimo La Rosa, Giuseppe Di Fatta, Salvatore Gaglio, Riccardo Rizzo, Alfonso Urso

**Affiliations:** 1 ICAR-CNR, National Research Council of Italy, Viale delle Scienze, ed.11, 90128 Palermo, Italy; 2 ICAR-CNR, National Research Council of Italy, via P. Castellino, 111, 80131 Napoli, Italy; 3 School of Systems Engineering, University of Reading, RG6 6AX, Reading, UK

**Keywords:** Molecular compounds, Self organizing map, Clustering, Visualization, Taverna

## Abstract

**Background:**

In many experimental pipelines, clustering of multidimensional biological datasets is used to detect hidden structures in unlabelled input data. Taverna is a popular workflow management system that is used to design and execute scientific workflows and aid in silico experimentation. The availability of fast unsupervised methods for clustering and visualization in the Taverna platform is important to support a data-driven scientific discovery in complex and explorative bioinformatics applications.

**Results:**

This work presents a Taverna plugin, the Biological Data Interactive Clustering Explorer (BioDICE), that performs clustering of high-dimensional biological data and provides a nonlinear, topology preserving projection for the visualization of the input data and their similarities. The core algorithm in the BioDICE plugin is Fast Learning Self Organizing Map (FLSOM), which is an improved variant of the Self Organizing Map (SOM) algorithm. The plugin generates an interactive 2D map that allows the visual exploration of multidimensional data and the identification of groups of similar objects. The effectiveness of the plugin is demonstrated on a case study related to chemical compounds.

**Conclusions:**

The number and variety of available tools and its extensibility have made Taverna a popular choice for the development of scientific data workflows. This work presents a novel plugin, BioDICE, which adds a data-driven knowledge discovery component to Taverna. BioDICE provides an effective and powerful clustering tool, which can be adopted for the explorative analysis of biological datasets.

## Background

The increasingly large amount of data related to DNA, proteins, molecular compounds, gene expressions and other biological sciences, raises the need for advanced analytical tools to support a data-driven scientific discovery. Classification and clustering of high-dimensional data, for example, are very popular techniques for the analysis of large multidimensional biological datasets [1]. Classification methods are based on a supervised learning approach, where the patterns of the training set belong to pre-defined classes. Dissimilarly, clustering algorithms use an unsupervised learning approach to find groups of similar data objects with no pre-defined classes. Clustering is typically used as an explorative tool. However, the interpretation of the clustering outcomes is often difficult and not intuitive, especially for large datasets with complex topological structures. For this reason, a suitable visualization method is a desirable tool to complement clustering analysis. Several methods (e.g., [2,3]) have been proposed to generate a visualisation of the outcomes of classification and clustering algorithms. The Self-Organizing Map (SOM) [4] is one of the best known unsupervised methods for data visualization and clustering [5,6]. SOM is an artificial neural network that generates a lattice of neurons, typically organized in a 2D grid, where high-dimensional data objects are projected to a lower dimensionality space while preserving their topological relations. SOMs are often used to generate clusters of similar data objects, but they can also be used to create 2D maps to facilitate the visual inspection of the relations induced by the adopted similarity function. Moreover, new data objects can be projected on a previously trained map in order to unveil similarities with other input data objects and performing a classification over the learned clusters.

In many experimental pipelines and workflows related to genomics, proteomics and other "omics" disciplines, the clustering and visualization of large multidimensional datasets is often used to identify and investigate similarities among data elements before further tests and analysis are carried out. The Taverna workbench [7] is one of the most popular tools to manage scientific workflows, especially in the bioinformatics domain. Taverna provides a broad range of components, which can be executed by local processors or Web services. It includes components to integrate remote resources, online databases and external analysis tools into user-defined workflows and can be extended with additional components by means of a service plug-in architecture.

This work presents a novel Taverna plug-in, the Biological Data Interactive Clustering Explorer (BioDICE), that performs clustering and provides visualization of biological datasets. The core algorithm in BioDICE is Fast Learning SOM (FLSOM) [8], an improved version of the classic SOM algorithm. FLSOM belongs to the category of the so-called Emergent Self Organizing Maps (ESOM) [9]. ESOMs have a number of neurons much larger than the number of input patterns to facilitate the discovery of emergent structures in the data. These structures are used to visualize multidimensional data objects and to identify clusters of similar objects by means of the U-Matrix visualization technique [2].

While there are a few SOM implementations available in Taverna, BioDICE fills a gap, as it is the first Taverna component performing SOM clustering with U-Matrix visualization.

RapidMiner is a data mining workflow execution engine and the RapidMiner plug-in [10] integrates RapidMiner operators into the Taverna environment. Although one of these operators provides an implementation of the classic SOM algorithm for dimensionality reduction, it does not generate a clustering model. There is another SOM implementation in RapidMiner that generates projections with U-Matrix and other visualization techniques. However, these visualization techniques generate static maps, do not allow an interactive exploration and do not detect clusters in the map automatically. Moreover, the RapidMiner plug-in for Taverna was based on RapidAnalytics, a server-based data mining workflow execution engine, which is no longer supported.

Another Taverna plugin that provides some machine learning services is the Chemistry Development Kit (CDK) plugin [11]. It integrates five clustering algorithms from the Weka machine learning library [12], which do not include SOM clustering.

This work introduces the BioDICE plug-in for Taverna: BioDICE is a pipeline of algorithms that performs a fast SOM clustering and generates a visualization of high-dimensional datasets. BioDICE supports an interactive generation of data partitions that represent emerging clusters.

## Implementation

**Figure 1:**
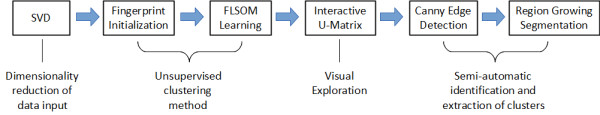
**BioDICE diagram block.** This diagram shows the six components of the BioDICE plugin.

BioDICE is composed by six main components, that are executed in cascade. Figure 1 shows the BioDICE block diagram. Biological data are given as input file in a tabular format as a list of objects with numerical features. The first component (*step 1*) is a dimensionality reduction operation, which applies the truncated Singular Value Decomposition (SVD) algorithm to the data vectors and maps the input space into a projected space of lower dimensionality. The most significant directions (singular vectors) are selected and used for the fingerprint initialization (*step 2*). In *step 3*, the FLSOM algorithm performs a clustering of the fingerprints and generates a U-Matrix visualization [2]. In *step 4*, an interactive view of the map is generated, with which the user can select data objects and investigate their similarities. A "Find Clusters" functionality allows the detection and refinement of data clusters in a semi-automatic way. In *step 5*, a customized version of the canny edge detection algorithm is applied to the 2D map. In *step 6*, a region growing algorithm generates a segmentation of the 2D map into disjoint partitions (clusters), using the boundaries provided by the edge detection algorithm.

In the following sections, the initialization and learning phases of FLSOM are discussed in more detail.

### Map Initialization

The output generated by a SOM is influenced by the initialization of the neuron weights. In BioDICE, a linear initialization technique [13] is adopted to improve the clustering results and to reduce the execution time. In general, linear initialization procedures are based on the analogy between SOMs and principal curve analysis algorithm, which is a non-linear generalization of the Principal Component Analysis (PCA). In BioDICE, the singular vectors obtained by SVD are used in place of the principal components to imprint the initial SOM lattice with fingerprints of the input objects. This initialization technique facilitates the learning process to converge towards a better clustering and faster than a random initialization.

### FLSOM learning

FLSOM provides an advanced SOM learning phase. A simulated annealing heuristic is combined with a standard batch SOM learning algorithm to obtain an adaptive learning rate [8]. This optimization technique improves both the quality and the convergence rate of the learning process. In FLSOM, the simulated annealing "temperature" is the Quantization Error (*QE*), which is defined as the average Euclidean distance between data vectors and their best matching units at the end of each learning epoch. The variation of *QE* (Δ_*QE*_) between two consecutive epochs is used to adapt the learning factor and, consequently, the convergence rate of the algorithm. The learning process stops when the value of Δ_*QE*_ is less than a user-defined threshold value. FLSOM was compared with other SOM-based algorithms, using both artificial and real biological datasets [8,14]. FLSOM provided a good convergence time and, most importantly, better results with respect to local distortion, topology preservation and clustering quality.

### User interface

The BioDICE user interface is composed by two panels: the configuration panel and the interactive map.

**Figure 2:**
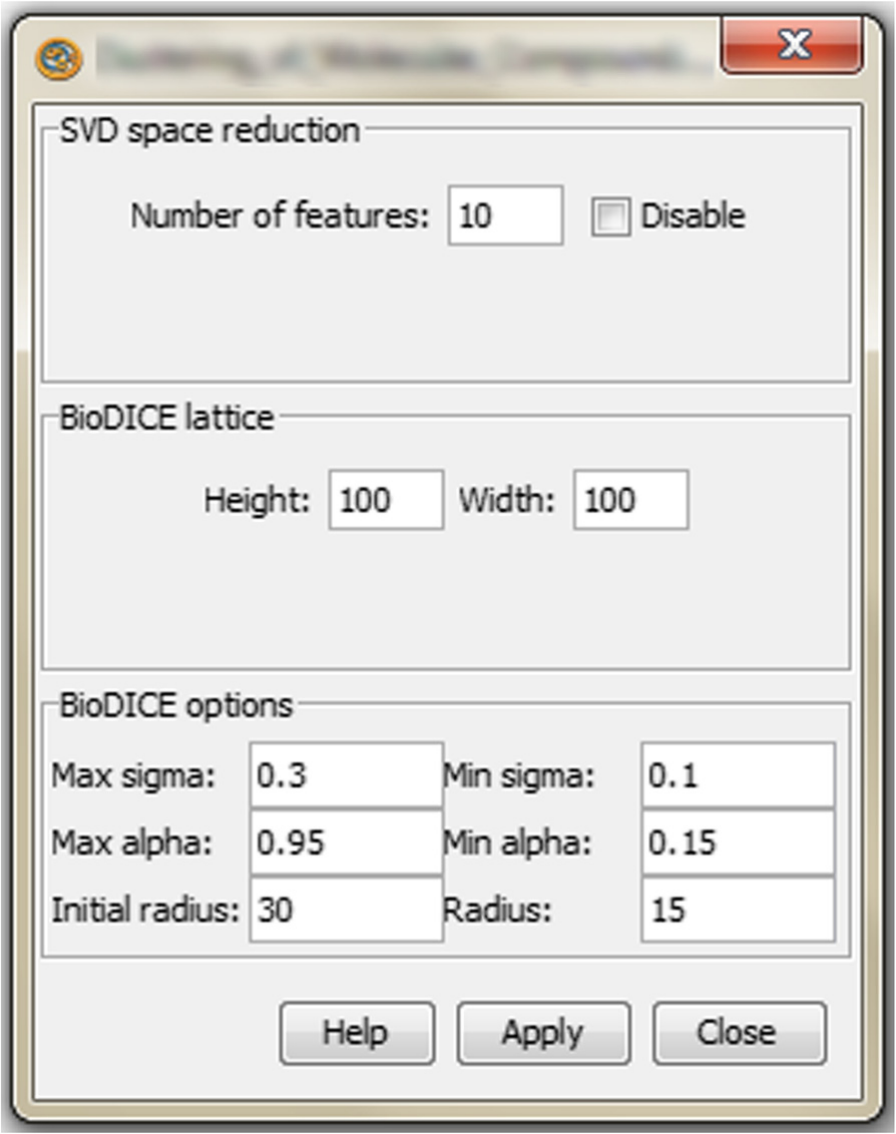
**BioDICE configuration panel.** This panel is used for setting the BioDICE parameters.

The configuration panel is available in the design perspective of Taverna from the BioDICE pop-up menu. It includes some user-defined parameters for setting the reduced dimensionality of the vector space (truncated SVD), the dimensionality of the lattice (map resolution) and the FLSOM learning parameters. A screenshot of this configuration panel is shown in Figure 2: the top panel contains the reduced dimensionality of the vector space, the centre panel contains the horizontal and vertical dimensions of the lattice and the bottom panel contains the FLSOM parameters (see [8] for further details).

**Figure 3:**
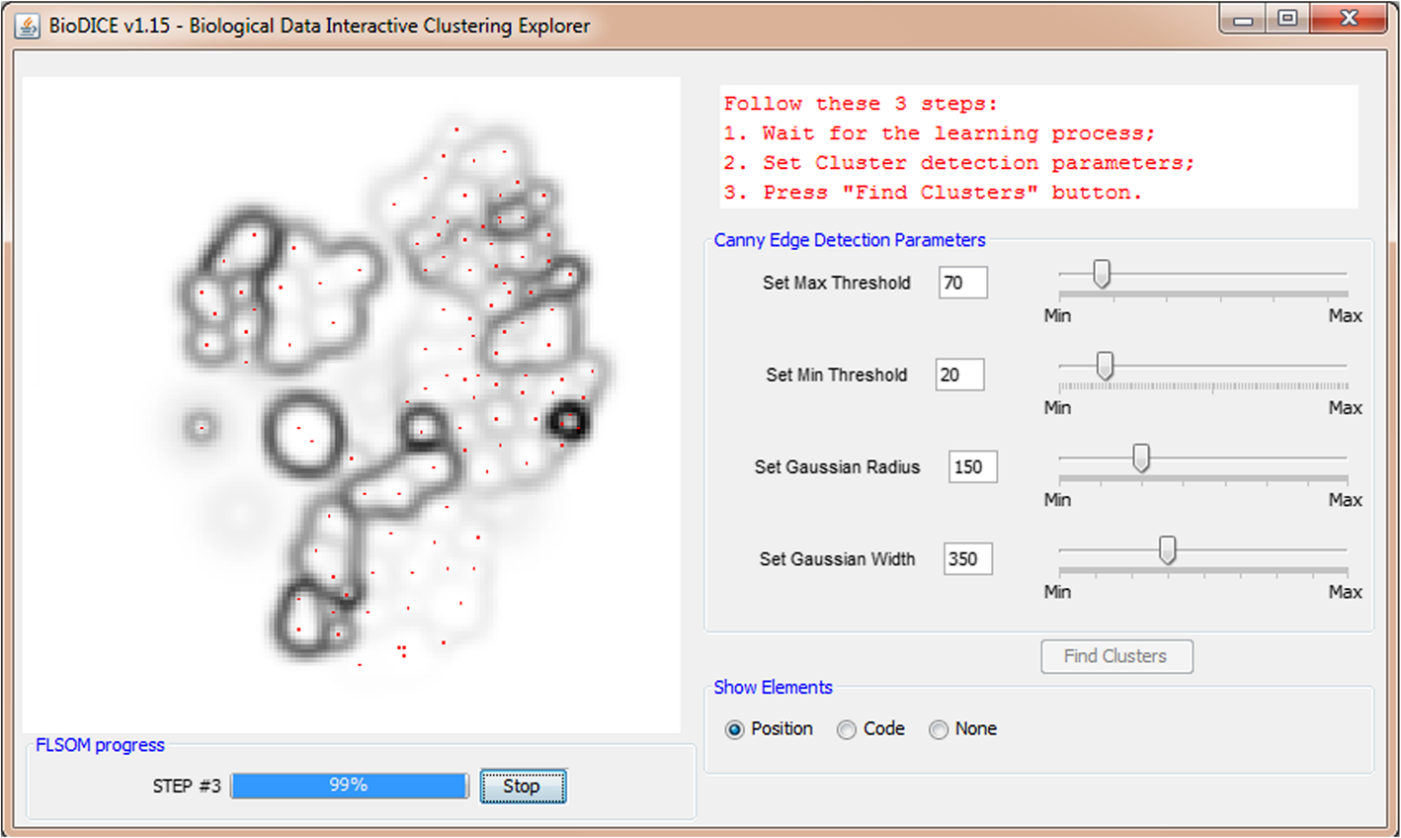
**BioDICE view.** This view shows the SOM lattice evolution during the learning process. At the end of the learning process, the view allows the interactive exploration of the input data and the extraction of clusters.

The view of the interactive map is available in the result perspective of Taverna, when the BioDICE plugin is running. It shows the progress of the learning process and the outcome, which is the U-Matrix representation of the SOM lattice. At the end of the learning process, the map becomes interactive and allows the exploration of the data objects and the detection of clusters of similar data objects. A screenshot of this view is shown in Figure 3. The interactive FLSOM U-Matrix map is on the left part of the view. The right part of the view contains four sliders, one for each parameter of the canny edge detection algorithm, and a button for the execution of the segmentation (*steps 5 and 6*) and the generation of the partitions (clusters).

## Results and discussion

In this Section, an application of the BioDICE plugin for the analysis of molecular compounds is presented. The analysis of similarities among chemical structures is an important challenge in cheminformatics [15,16]: the main goal is to gain understanding about the relationship of the compounds with respect to their chemical or functional activity. In previous works [6,8], the FLSOM algorithm was shown to be very effective in the cluster analysis of molecular compounds.

The BioDICE Taverna plugin requires an input file containing a *features* ×*patterns* data table, that is a matrix with the feature identifiers as rows and the chemical compound identifiers as columns. The plugin also accepts two optional inputs, which are used for enriching the graphical representation of the chemical compounds: an ordered list of input compounds and a list of their corresponding representations in *SMILES* notation.

**Figure 4:**
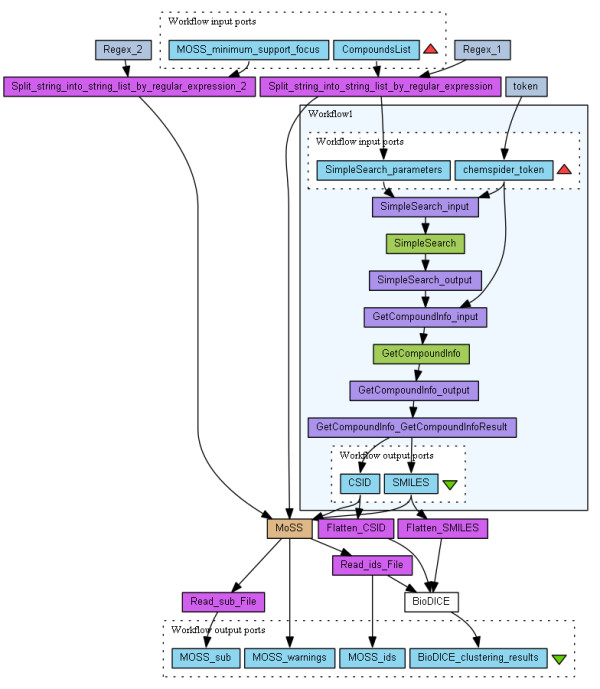
**Taverna workflow for a cheminformatics scenario.** The workflow downloads a list of chemical compounds from ChemSpider database in *SMILES* format. A set of frequent fragments are generated as features of the compounds. The BioDICE plugin performs clustering and generates an interactive map.

The whole Taverna workflow for this case study is shown in Figure 4. A videotutorial for installation and execution of this case study is available at https://www.youtube.com/watch?v=eKpf-K2T8hw. At execution, the workflow retrieves a set of molecular compounds in *SMILES* format from the ChemSpider database [17], associated to a list of compound names (or identifiers) as input. The selected compounds are then processed using MoSS [18], a frequent subgraph mining algorithm. MoSS extracts a set of frequent molecular fragments contained in the input set of molecular compounds. The extracted fragments are used as features of the input compounds: MoSS generates the *features* × *patterns* matrix, which has the fragment identifiers as rows and the compound identifiers as columns. BioDICE is then used to process the *features* × *patterns* matrix to detect and visualize clusters of molecular compounds, where similarity is computed with respect to the frequent fragments. If the data matrix has too many features, BioDICE can reduce the dimensionality of the features space using a truncated SVD, thus mitigating the curse of dimensionality.

The workflow of Figure 4 contains two nested Taverna workflows, which have been made publicly available and can be retrieved from the repository *myExperiment*[19] at http://www.myexperiment.org/workflows/1412.html and http://www.myexperiment.org/workflows/1427.html. The complete workflow of Figure 4 can be retrieved at http://www.myexperiment.org/workflows/3611.html.

**Figure 5:**
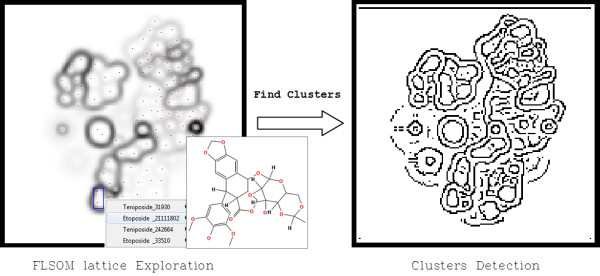
**Trained map and cluster detection.** The map (left) shows the input data objects within regions of similarity (light areas). Dark boundaries divide regions with dissimilar data objects. The map is interactive: an area selection operation generates the list of compounds in the area. The boundaries of the clusters (right) are obtained with a Canny filter and an image segmentation algorithm. The Canny filter can be tuned in order to adjust the clusters detection interactively.

The workflow has been tested with a sample data set obtained from the NCI DTP Discovery Service [20], a set of 101 FDA-approved anticancer drugs. The resulting BioDICE 2D map is shown in the left part of Figure 5 with a typical U-Matrix visualization. The user can interact with the map, selecting an area and obtaining this way the list of elements belonging to that part. The right part of Figure 5 shows the detected boundaries of the clusters as generated by the Canny filter and region segmentation. A complete list of the compounds in each cluster is generated by means of the "Find Cluster" button and is stored as a text file.

## Conclusions

BioDICE is a new plugin for the Taverna workbench, that can be adopted to perform fast clustering of multidimensional biological datasets and to generate their interactive visualization. BioDICE is based on the FLSOM algorithm, an improved version of SOM learning algorithm. An application scenario in cheminformatics has been discussed to demonstrate the use of the plugin. A dataset of molecular compounds in *SMILES* format has been first processed with a frequent subgraph mining algorithm (feature generation). BioDICE has been applied to provide a cluster analysis of the compounds with respect to the extracted features. BioDICE has generated an interactive map of the input compounds and a list of the compounds in each detected cluster.

The BioDICE plugin, the documentation, a tutorial (covering installation, configuration and use), the workflow and the dataset used in this work are available at http://biolab.pa.icar.cnr.it/biodice.html.

## Availability and requirements

• **Project name:** BioDICE

• **Project home page:**http://biolab.pa.icar.cnr.it/biodice.html

• **Operating system(s):** Platform independent

• **Programming language:** Java

• **Other requirements:** Java 1.6 or higher, Taverna 2.3.0. For compatibility issues between Java runtime version and MoSS tool please refer to our project home page.

• **License:** GNU GPL v3

• **Any restrictions use by non-academics:** Only those imposed already by the license

## Competing interests

The authors declare that there are no competing interests.

## Authors’ contributions

AF: project conception, FLSOM implementation, plugin implementation, experimental tests, writing, assessment, discussions. MLR: project conception, plugin implementation, experimental tests, writing, assessment, discussions. GDF: project conception, writing, discussions. SG: project conception, discussions. RR: assessment, discussions. AU: assessment, discussions, funding. All authors read and approved the final manuscript.
